# Supplementary material for: Microglial metabolic flexibility supports immune surveillance of the brain parenchyma
Source: Nat Commun. 2020 Mar 25;11:1559. doi: 10.1038/s41467-020-15267-z (PMC7096448; doi:10.1038/s41467-020-15267-z)
Supplement: Supplementary file 1 — Supplementary Information [file 41467_2020_15267_MOESM1_ESM.pdf]

**Supplementary Information**

# Microglial metabolic flexibility supports immune surveillance of the brain parenchyma.

Bernier et al.

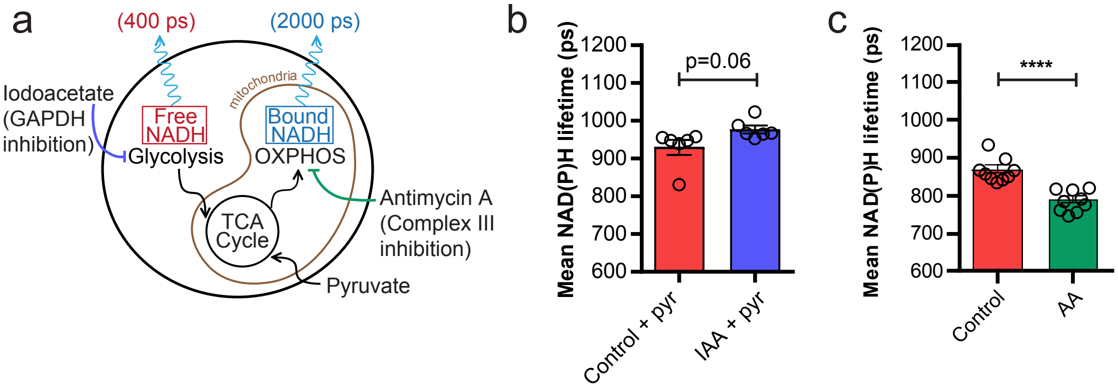


**Supplementary figure 1. Mean NAD(P)H lifetime as a measure of cellular metabolism.** *Related to figures 1, 4, and 6.*

**a,** Schematic representation of inhibitors Iodoacetate (IAA) and Antimycin A (AA), and predicted impacts on NAD(P)H lifetime. **b,** Mean NAD(P)H lifetime of microglia after a 30-minute incubation in aCSF with or without 50 µM Iodoacetate in the presence of exogenously added 2 mM pyruvate (pyr) to act as an alternative carbon supply (p=0.06 by unpaired, two-tailed t-test; n=6 slices). **c,** Mean NAD(P)H lifetime of microglia after a 30-minute incubation in aCSF with or without Antimycin A (4 µM; ****, p=0.0000281 by unpaired, two-tailed t-test; n= 9 slices). Source data are provided as a Source Data file. Data are represented as mean ± SEM.

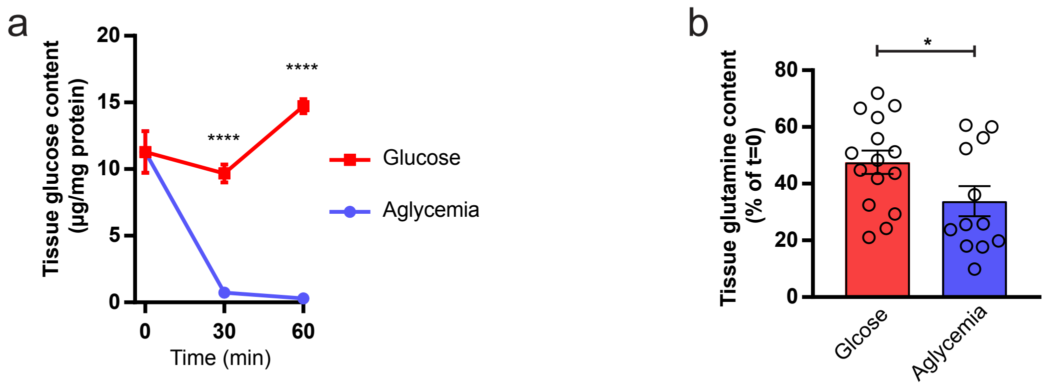


**Supplementary figure 2. Absence of glucose, but presence of glutamine in brain tissue during aglycemia treatment.** *Related to figures 3 to 9.*

**a,** In acute brain slices, glucose within the tissue was measured at indicated time-points during a one-hour incubation in normal glucose-containing aCSF or in aglycemic aCSF. Note that the control (glucose) slices were rinsed in glucose-free aCSF prior to tissue glucose measurement to ensure glucose is not carried directly from the incubation bath aCSF. (n= 3 mice, 15 slices; 30 min: ****, p=0.00009; 60 min: ****, p=0.0000007, two-way ANOVA, Bonferroni post-test). **b,** In acute brain slices, glutamine content was measured after a one-hour incubation in normal glucose-containing aCSF or in aglycemic aCSF. Shown is the remaining glutamine content relative to time 0 (onset of incubation), in %. (n= 6 mice, 15 slices for glucose, 12 slices for aglycemia; *, p=0.0496 by unpaired, two-tailed t-test). Data are represented as mean ± SEM. Source data are provided as a Source Data file.

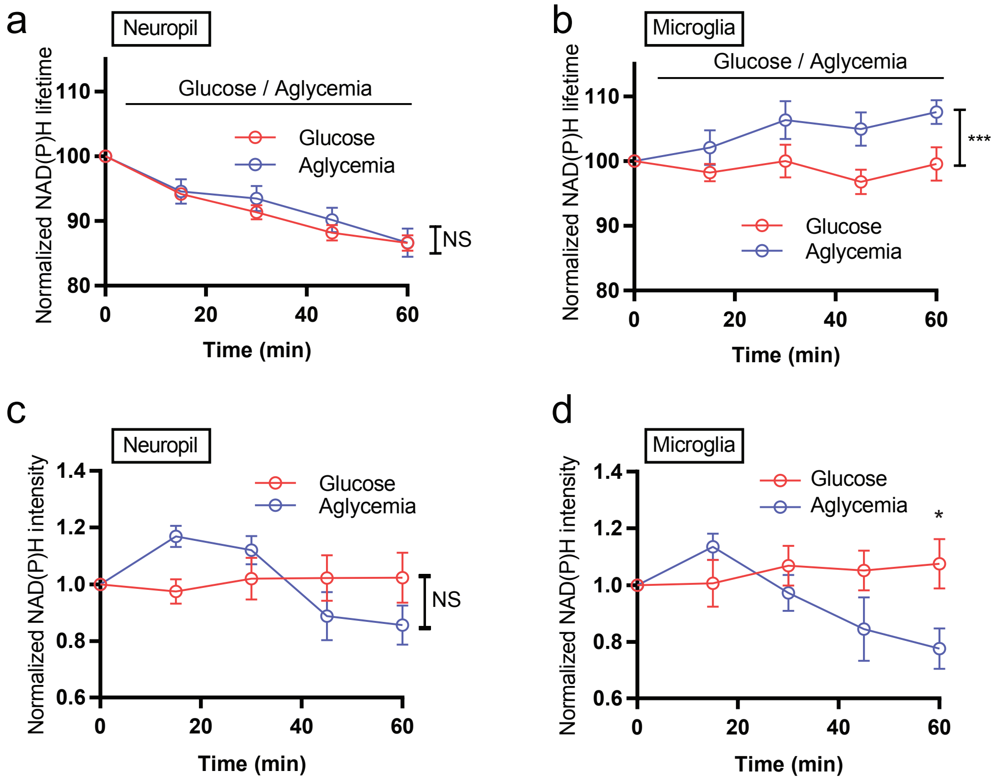


**Supplementary figure 3. NAD(P)H fluorescence lifetime and intensity in situ during aglycemia.** *Related to figure 4.*

**a,** Mean NAD(P)H lifetime of neuropil during 60 minutes in either glucose-containing aCSF or aglycemic aCSF (Normalized to t=0; n= 4 mice, 12 neuropil ROIs for glucose, 11 for aglycemia; NS, $p>0.05$, two-way ANOVA). **b,** Mean NAD(P)H lifetime of microglia during 60 minutes in either glucose-containing aCSF or aglycemic aCSF (Normalized to t=0; n= 4 mice, 12 microglia for glucose, 11 for aglycemia; ***, $p=0.0001$, treatment factor, two-way ANOVA). **c,** NAD(P)H fluorescence intensity of neuropil during 60 minutes in either glucose-containing aCSF or aglycemic aCSF, normalized to t=0 (n= 4 mice, 12 neuropil ROIs for glucose, 11 for aglycemia; NS, $p>0.05$, two-way ANOVA). **d,** NAD(P)H fluorescence intensity of microglia during 60 minutes in either glucose-containing aCSF or aglycemic aCSF, normalized to t=0 (n= 4 mice, 12 microglia for glucose, 11 for aglycemia; *, $p=0.0322$, repeated analysis, two-way ANOVA). Data are represented as mean ± SEM. . Source data are provided as a Source Data file.

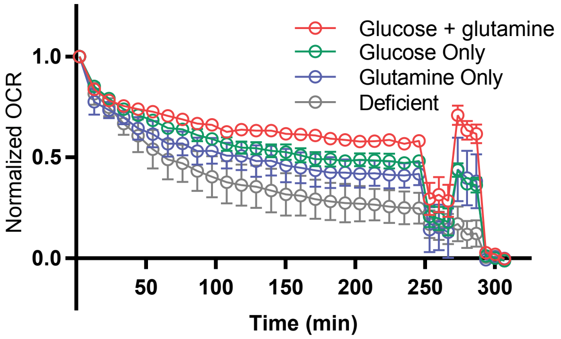


**Supplementary figure 4. Microglial mitochondrial metabolism *in vitro*.** *Related to figure 5.*

Oxygen consumption rate (OCR) of primary microglia during a Seahorse Extracellular Flux Analyzer mitochondrial stress test including a 4-hour pre-treatment in control (glucose+glutamine), glucose-only, glutamine-only or deficient (no glucose, no glutamine) media (n= 3 experimental trials (Seahorse plates), wells/condition: 19 control; 24 glucose only; 24 glutamine only; 23 deficient). Data in each well is normalized to its initial OCR reading. Data are represented as mean ± SEM. Source data are provided as a Source Data file.

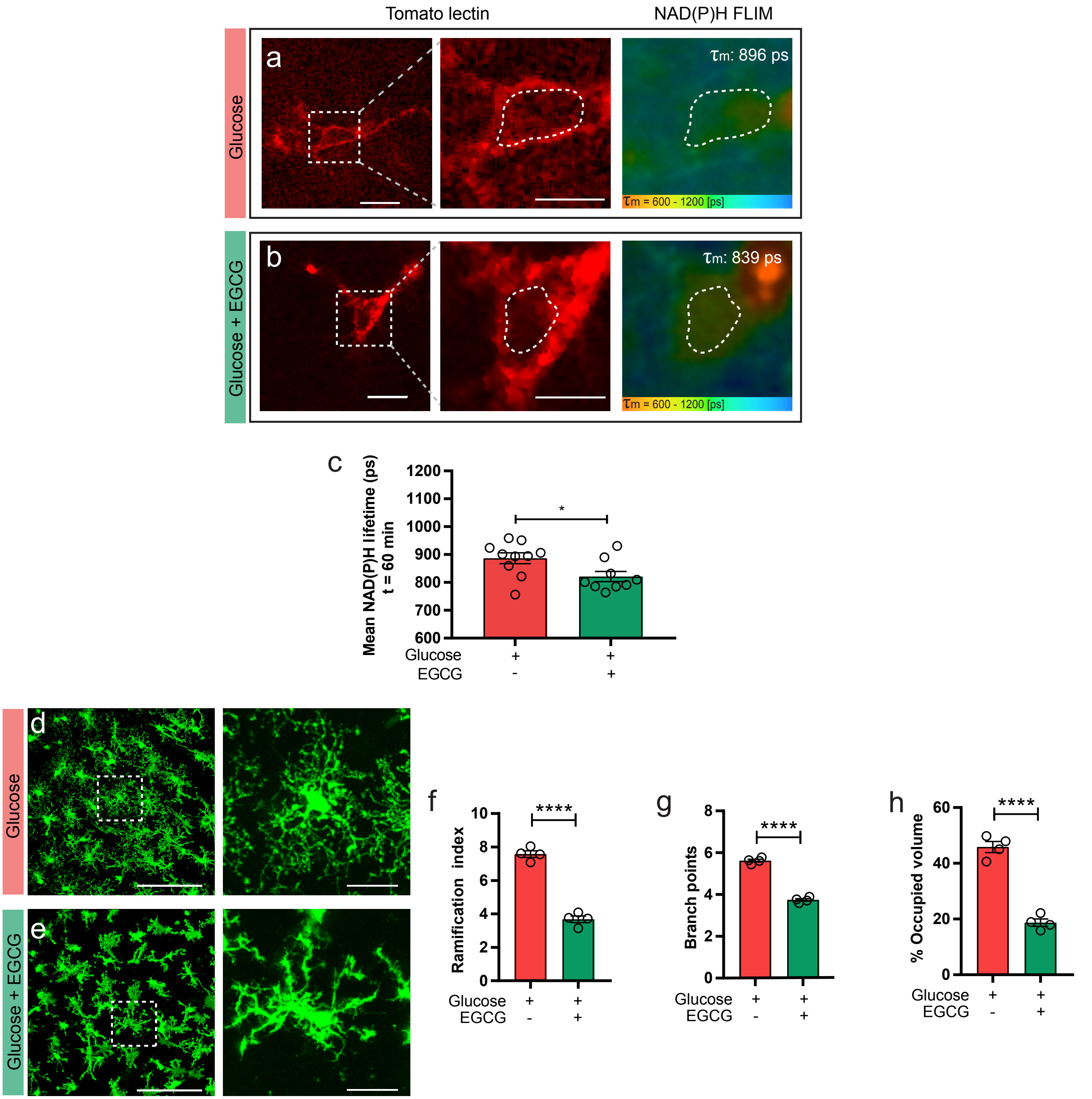


**Supplementary figure 5. Role of microglial glutaminolysis in the presence of glucose.** *Related to figure 6 and 7.* **a,b,** Representative images of FLIM-NAD(P)H imaging of microglia labelled with tomato lectin, following a 60-minute incubation in glucose aCSF **(a)** or glucose aCSF with EGCG **(b)**. Indicated on the right panel is the mean NAD(P)H lifetime of the microglial ROI within the dotted line (for **a-c**, n= 3 mice, 10 microglia for glucose, n= 3 mice, 9 microglia for glucose+EGCG). **c,** Mean NAD(P)H lifetime of microglia after a 60-minute incubation in glucose-containing aCSF, with or without EGCG (100 µM; *, p= 0.0248, unpaired, two-tailed t-test). **d,e,** Microglial morphology identified in CX3CR1EGFP/+ acute brain slice following a 60-minute incubation in glucose aCSF **(d)** or glucose aCSF with EGCG **(e)** (for panel **d-h**, glucose: n= 4 mice, 8 slices, 141 cells; glucose+EGCG: n= 4 mice, 8 slices, 142 cells). **f,** Ramification index of microglia 60 minutes after glucose or glucose+EGCG incubation (****, p=0.000008, unpaired, two-tailed t-test). **g,** Number of branch points per microglia (****, p=0.000001, unpaired, two-tailed t-test). **h,** Percentage of brain volume occupied by microglia (****, p=0.000028, unpaired, two-tailed t-test). Data are represented as mean ± SEM. Scale bars, 10 µm (**a,b** left panel); 5 µm (**a,b** middle panel); 100 µm (**d,e** left panel); 20 µm (**d,e** right panel). Source data are provided as a Source Data file.

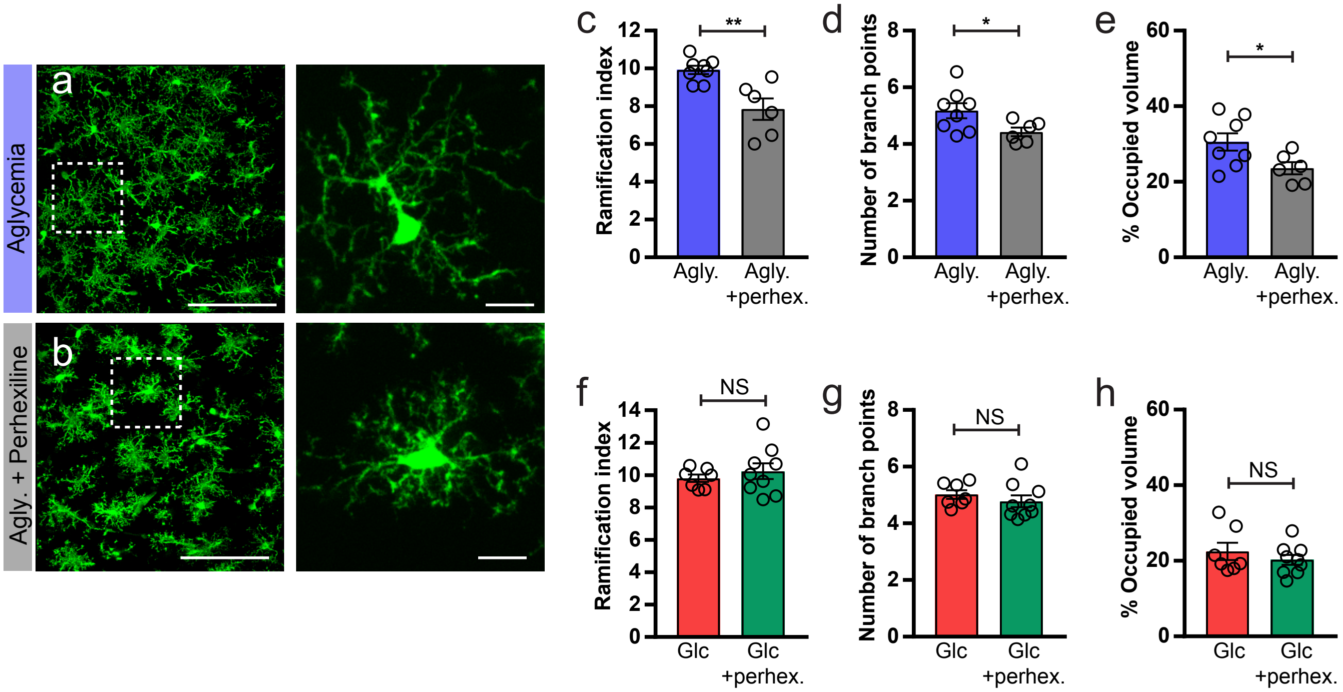


**Supplementary figure 6. CPT1 inhibition by perhexiline during aglycemia reduces microglial ramification.** *Related to figure 8.*

**a,b,** Microglial morphology following a 60-minute incubation in aglycemic aCSF **(a)** or aglycemic aCSF with perhexiline **(b)**. **c,** Ramification index of microglia 60 minutes after aglycemia or aglycemia+perhexiline incubation (**, p= 0.0028, unpaired, two-tailed t-test; for panels **c-h**, glucose: n= 4 mice, 7 slices, 107 cells; glucose+perhexiline: n= 4 mice, 9 slices, 99 cells; aglycemia: n= 4 mice, 8 slices, 182 cells; aglycemia+perhexiline: n= 4 mice, 7 slices, 158 cells). **d,** Number of branch points per microglia (*, p= 0.0457, unpaired, two-tailed t-test). **e,** Percentage of brain volume occupied by microglia (*, p= 0.0389, unpaired, two-tailed t-test). **f,** Ramification index of microglia 60 minutes after glucose or glucose+perhexiline incubation (NS, p>0.05, unpaired, two-tailed t-test). **g,** Number of branch points per microglia (NS, p>0.05, unpaired, two-tailed t-test). **h,** Percentage of brain volume occupied by microglia (NS, p>0.05, unpaired, two-tailed t-test). Data are represented as mean ± SEM. Scale bars, 100 µm (**a,b** left panel); 20 µm (**a,b** right panel). Source data are provided as a Source Data file.

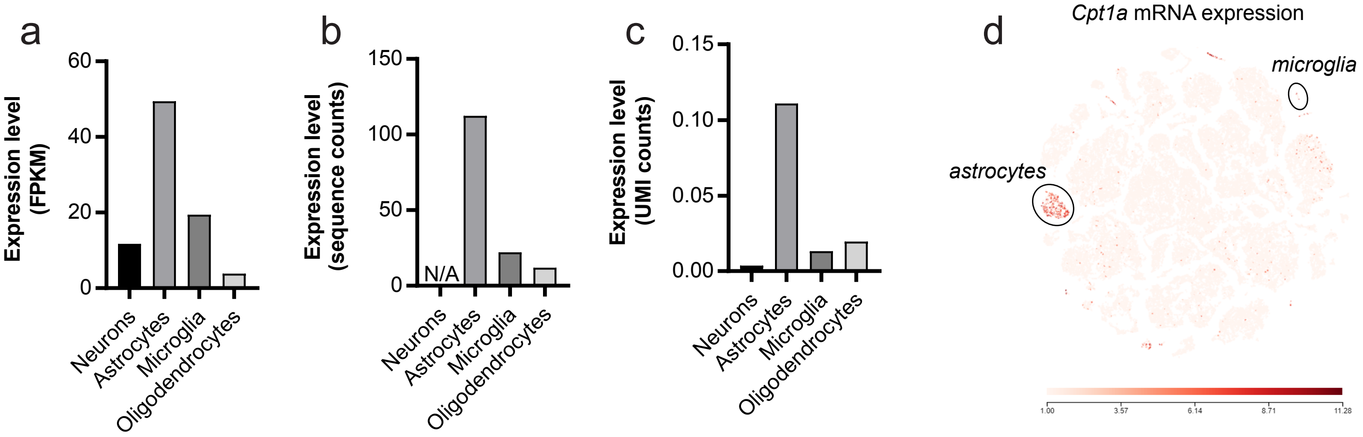


**Supplementary figure 7. Brain *Cpt1a* transcription is mostly observed in astrocytes.** *Related to figure 8.* *Cpt1a* mRNA transcription level based on the publicly available transcriptomes from (**a**) 'Brain RNA-Seq' by the Barres lab [Zhang et al (2014) DOI: https://doi.org/10.1523/JNEUROSCI.1860-14.2014], (**b**) http://betsholtzlab.org/VascularSingleCells/database.html by the Betsholtz lab [Vanlandewijck, M., He, L. et al. Nature, 554, 475-480 (2018); He, L., Vanlandewijck, M. et al. Scientific Data, Volume 5, Article number: 180160 (2018)], and (**c**) Mousebrain.org by the Linnarsson lab [Zeisel A et al., Cell, 2018 Aug 9;174(4):999-1014.e22]. **d,** Transcription of *Cpt1a* mRNA in single cells clustered by t-SNE analysis, available from the Allen Brain Atlas [Allen Cell Types Database (2015); https://celltypes.brain-map.org/r-naseq/mouse/cortex-and-hippocampus]. Source data are provided as a Source Data file.

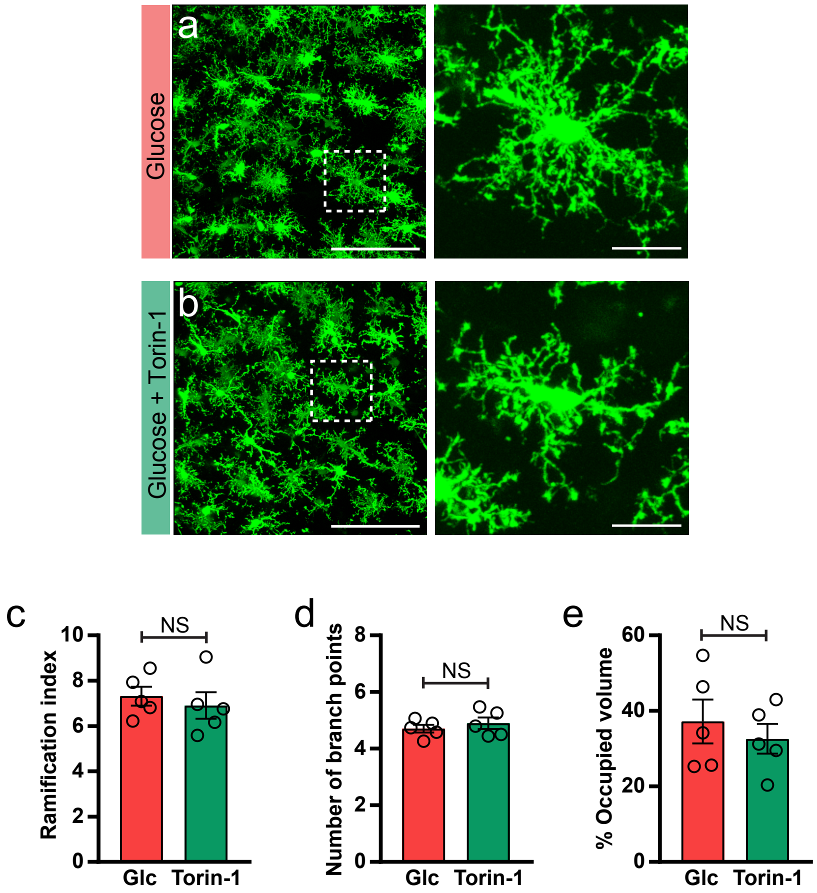


**Supplementary figure 8. mTOR inhibition has no effect of microglial surveillance in the presence of glucose.** *Related to figure 9.*

**a,b,** Microglial morphology following a 60-minute incubation in glucose aCSF **(a)** or glucose aCSF with Torin-1 (b, 10 μM). **c,** Ramification index of microglia 60 minutes after glucose or glucose+Torin-1 incubation (NS, $p>0.05$, unpaired, two-tailed t-test; for panels **c-e,** glucose: n= 5 mice, 9 slices, 195 cells; glucose+Torin-1: n= 5 mice, 8 slices 178 cells). **d,** Number of branch points per microglia (NS, $p>0.05$, unpaired, two-tailed t-test). **e,** Percentage of brain volume occupied by microglia (NS, $p>0.05$, unpaired, two-tailed t-test). Data are represented as mean ± SEM. Scale bars,100 μm (**a,b** left panel); 20 μm (**a,b** right panel). Source data are provided as a Source Data file.
