# Supplementary material for: Microglial metabolic flexibility supports immune surveillance of the brain parenchyma
Source: Nat Commun. 2020 Mar 25;11:1559. doi: 10.1038/s41467-020-15267-z (PMC7096448; doi:10.1038/s41467-020-15267-z)
Supplement: Supplementary file 3 — Description of Additional Supplementary Information [file 41467_2020_15267_MOESM3_ESM.pdf]

Description of Additional Supplementary Files

File Name: Supplementary Movie 1
Description: Microglial process motility and damage-sensing during insulin-induced hypoglycemia. *Related to figure 2.* Time lapse video of cortical microglia from $CX3CR1^{EGFP/+}$ obtained with two-photon microscopy *in vivo* through a cranial window. Microglial surveillance of the brain parenchyma and response to focal damage are unaltered by insulin injection and subsequent hypoglycemia. See Fig. 2c for equivalent timeline of glucose concentration decrease. Duration: 100 minutes. 1 minute per frame. Insulin (5 U/kg) injected at minute 15. Laser-induced lesion at minute 75. 128 µm X 128 µm.

File Name: Supplementary Movie 2
Description: Microglial process motility in the absence of glucose. *Related to figure 3.*
Time lapse video of hippocampal microglia from $CX3CR1^{EGFP/+}$ obtained with two-photon microscopy *in situ* in acute brain slices. Microglial surveillance of the brain parenchyma is unaltered in the complete absence of glucose. Duration: 75 minutes. 1 minute per frame. aCSF was switched to aglycemic aCSF at minute 15. 110 µm X 118 µm.

File Name: Supplementary Movie 3
Description: Microglial process convergence to lesion damage in normoglycemia. *Related to figure 3.* Time lapse video of hippocampal microglia from $CX3CR1^{EGFP/+}$ obtained with two-photon microscopy *in situ* in acute brain slices. Microglia extend their processes towards a local laser-induced lesion in normoglycemic conditions. Duration: 30 minutes. 1 minute per frame. Focal damage was induced at minute 15. 144 µm X 144 µm.

File Name: Supplementary Movie 4
Description: Microglial process convergence to lesion damage during aglycemia. *Related to figure 3.* Time lapse video of hippocampal microglia from $CX3CR1^{EGFP/+}$ obtained with two-photon microscopy *in situ* in acute brain slices. Microglia extend their processes towards a local laser-induced lesion following a 75-minute incubation in glucose-free conditions. Duration: 30 minutes. 1 minute per frame. Focal damage was induced at minute 15 (90 minutes after aglycemia onset). 144 µm X 144 µm.

File Name: Supplementary Movie 5
Description: Microglial process motility is inhibited by EGCG during aglycemia. *Related to figure 7.*
Time lapse video of hippocampal microglia from $CX3CR1^{EGFP/+}$ obtained with two-photon microscopy *in situ* in acute brain slices. Microglial process motility is inhibited by addition of EGCG to block glutaminolysis during aglycemic conditions. Duration: 135 minutes. 1 minute per frame. aCSF was switched to aglycemic aCSF at minute 10, then EGCG (100 µM) was added at minute 90. 134 µm X 140 µm.

File Name: Supplementary Movie 6
Description: Microglial process convergence to lesion during aglycemia is inhibited by EGCG. *Related to figure 7.* Time lapse video of hippocampal microglia from $CX3CR1^{EGFP/+}$ obtained with two-

photon microscopy *in situ* in acute brain slices. Slices were incubated in aglycemic aCSF + EGCG (100 µM) for 75 minutes, then imaged. The presence of EGCG in aglycemia inhibited the microglial response to local damage. Duration: 30 minutes. 1 minute per frame. Focal damage was induced at minute 15 (90 minutes after aglycemia + EGCG). 136 µm X 136 µm.

File Name: Supplementary Movie 7

Description: Microglial process convergence to lesion during aglycemia is inhibited by Torin-1. *Related to figure 9.* Time lapse video of hippocampal microglia from CX3CR1$^{EGFP/+}$ obtained with two-photon microscopy *in situ* in acute brain slices. Slices were incubated in aglycemic aCSF + Torin-1 (10 µM) for 75 minutes, then imaged. The presence of Torin-1 in aglycemia inhibited the microglial response to local damage. Duration: 20 minutes. 1 minute per frame. Focal damage was induced at the start of the video (90 minutes after aglycemia + Torin-1). 147 µm X 147 µm.
