# Supplementary material for: Microglial metabolic flexibility supports immune surveillance of the brain parenchyma
Source: Nat Commun. 2020 Mar 25;11:1559. doi: 10.1038/s41467-020-15267-z (PMC7096448; doi:10.1038/s41467-020-15267-z)
Supplement: Supplementary file 11 — Reporting summary [file 41467_2020_15267_MOESM11_ESM.pdf]

nature research

Corresponding author(s): Brian A. MacVicar

Last updated by author(s): Feb 14, 2020

Reporting Summary

Nature Research wishes to improve the reproducibility of the work that we publish. This form provides structure for consistency and transparency in reporting. For further information on Nature Research policies, see Authors & Referees and the Editorial Policy Checklist .

Statistics

For all statistical analyses, confirm that the following items are present in the figure legend, table legend, main text, or Methods section.

n/a	Confirmed	
☐	☒	The exact sample size (*n*) for each experimental group/condition, given as a discrete number and unit of measurement
☐	☒	A statement on whether measurements were taken from distinct samples or whether the same sample was measured repeatedly
☐	☒	The statistical test(s) used AND whether they are one- or two-sided<br>*Only common tests should be described solely by name; describe more complex techniques in the Methods section.*
☐	☒	A description of all covariates tested
☐	☒	A description of any assumptions or corrections, such as tests of normality and adjustment for multiple comparisons
☐	☒	A full description of the statistical parameters including central tendency (e.g. means) or other basic estimates (e.g. regression coefficient) AND variation (e.g. standard deviation) or associated estimates of uncertainty (e.g. confidence intervals)
☐	☒	For null hypothesis testing, the test statistic (e.g. *F*, *t*, *r*) with confidence intervals, effect sizes, degrees of freedom and *P* value noted<br>*Give P values as exact values whenever suitable.*
☒	☐	For Bayesian analysis, information on the choice of priors and Markov chain Monte Carlo settings
☒	☐	For hierarchical and complex designs, identification of the appropriate level for tests and full reporting of outcomes
☒	☐	Estimates of effect sizes (e.g. Cohen's *d*, Pearson's *r*), indicating how they were calculated

Our web collection on statistics for biologists contains articles on many of the points above.

Software and code

Policy information about availability of computer code

Data collection	Zen Imaging software, ScanImage (version 3.8), and Becker & Hickl SPCM and SPCImage (version 7.4) were used in data collection and analysis.
Data analysis	Fiji and 3DMorph (both freely available online) were used in data analysis. Custom MATLAB script was generated to measure microglia motility over time, which is described in detail in the Methods section of the manuscript. GraphPad Prism 7 was used to calculate statistical tests presented in this manuscript.

For manuscripts utilizing custom algorithms or software that are central to the research but not yet described in published literature, software must be made available to editors/reviewers. We strongly encourage code deposition in a community repository (e.g. GitHub). See the Nature Research guidelines for submitting code & software for further information.

Data

Policy information about availability of data

All manuscripts must include a data availability statement. This statement should provide the following information, where applicable:

- Accession codes, unique identifiers, or web links for publicly available datasets
- A list of figures that have associated raw data
- A description of any restrictions on data availability

A source data file is provided with all relevant raw data presented in the study. These correspond to figures 1d; 2b,c,d,e,f,i,k,l; 3e,f,g,i,j,m,n; 4d,e,f; 5a,b,c,d; 6c,d; 7e,f,g,j,k,l; 8e,f,g,j,k,l,m; 9e,f,g,j,k,l; and supplemental figures 1b,c; 2a,b; 3a,b,c,d; 4; 5c,f,g,h; 6c,d,e,f,g,h; 7a,b,c; 8c,d,e.

Field-specific reporting

Please select the one below that is the best fit for your research. If you are not sure, read the appropriate sections before making your selection.

- [x] Life sciences
- [ ] Behavioural & social sciences
- [ ] Ecological, evolutionary & environmental sciences

For a reference copy of the document with all sections, see nature.com/documents/nr-reporting-summary-flat.pdf

Life sciences study design

All studies must disclose on these points even when the disclosure is negative.

Sample size	Sample sizes were chosen to maximize certainty in experimental results, while also taking into consideration the ethical concerns of using mouse tissue. A minimum of three biological Ns were included per experiment and within each, multiple cells across several slices were measured. Given the data separation between groups and measurement sensitivity, these sample sizes were sufficient to distinguish biological effects, or a lack thereof.
Data exclusions	Only data points beyond two standard deviations from the mean were considered as outliers and excluded. These exclusion criteria were established prior to data collection and analysis, and were chosen to account for unexpected variability between experiments, which could alter results and interpretations.
Replication	All experiments were repeated multiple times (at a minimum of three) to ensure the observed result could be replicated. Furthermore, results were consistent in all cases where multiple researchers performed the same measurements.
Randomization	When possible, tissue from a single mouse was separated into all treatment conditions, therby providing its own internal control. Therefore, any individual variations will not specifically effect any particular group. Furthermore, mouse genotypes were kept consistent throughout experiments to minimize between-sample variabilities.
Blinding	This data was not blinded during collection or data analysis, as the researcher responsible for making measurements needed to be aware of any solution or pharmacological manipulations in order to conduct the experiment and process results appropriately. To avoid bias and variability between experimenters, detailed methods were laid out prior to data analysis, which were strictly followed.

Reporting for specific materials, systems and methods

We require information from authors about some types of materials, experimental systems and methods used in many studies. Here, indicate whether each material, system or method listed is relevant to your study. If you are not sure if a list item applies to your research, read the appropriate section before selecting a response.

Materials & experimental systems

n/a	Involved in the study	
x		Antibodies
	x	Eukaryotic cell lines
x		Palaeontology
	x	Animals and other organisms
x		Human research participants
x		Clinical data

Methods

n/a	Involved in the study	
x		ChIP-seq
x		Flow cytometry
x		MRI-based neuroimaging

Eukaryotic cell lines

Policy information about cell lines

Cell line source(s)	Rat E18 cortex primary cell preparation.
Authentication	Cells used were not authenticated. Rather, a previously established technique was used to optimize for cell type of interest.
Mycoplasma contamination	Cells were not tested for Mycoplasma contamination.
Commonly misidentified lines (See ICLAC register)	No commonly misidentified lines were used in this study.

Animals and other organisms

Policy information about studies involving animals; ARRIVE guidelines recommended for reporting animal research

Laboratory animals	For cell cultures, male and female Sprague Dawley rats were used at E18. For NAD(P)H lifetime imaging, male C57Bl/6 mice from 2-5 months of age were used. For all other imaging experiments, CX3CR1+/EGFP male and female mice of 2-12 months of age were included. All animals were housed in accordance with UBC and CCAC guidelines, on a 12 hour light-dark cycle, standard room temperature and humidity, and ad libitum access to food and water.
Wild animals	This study did not involve any wild animals.
Field-collected samples	This study does not include any samples collected from the field.
Ethics oversight	The Canadian Council on Animal Care set the national ethical standards for use of animal tissue, which was adhered to, and all protocols were further approved by the University of British Columbia committee on animal care.

Note that full information on the approval of the study protocol must also be provided in the manuscript.
